# Effect of psyllium and gum Arabic biopolymers on the survival rate and storage stability in yogurt of *Enterococcus durans* IW3 encapsulated in alginate

**DOI:** 10.1002/fsn3.430

**Published:** 2016-10-11

**Authors:** Yousef Nami, Babak Haghshenas, Ahmad Yari Khosroushahi

**Affiliations:** ^1^ Department of Food BiotechnologyBranch for Northwest & West regionAgricultural Biotechnology Research Institute of IranAgricultural ResearchEducation and Extension Organization (AREEO)TabrizIslamic Republic of Iran; ^2^ Institute of BiosciencesUniversity Putra MalaysiaSelangorMalaysia; ^3^ Drug Applied Research CenterFaculty of PharmacyTabriz University of Medical SciencesTabrizIslamic Republic of Iran; ^4^ Department of PharmacognosyFaculty of PharmacyTabriz University of Medical SciencesTabrizIslamic Republic of Iran

**Keywords:** alginate, encapsulation, *Enterococcus durans*, gum Arabic, psyllium

## Abstract

Different herbal biopolymers were used to encapsulate *Enterococcus durans* IW3 to enhance its storage stability in yogurt and subsequently its endurance in gastrointestinal condition. Nine formulations of encapsulation were performed using alginate (ALG), ALG‐psyllium (PSY), and ALG‐gum Arabic (GA) blends. The encapsulation efficiency of all formulations, tolerance of encapsulated *E. durans* IW3 against low pH/high bile salt concentration, storage lifetime, and release profile of cells in natural condition of yogurt were evaluated. Result revealed 98.6% encapsulation efficiency and 76% survival rate for all formulation compared with the unencapsulated formulation cells (43%). The ALG‐PSY and ALG‐GA formulations have slightly higher survival rates at low pH and bile salt condition (i.e., 76–93% and 81–95%, respectively) compared with the ALG formulation. All encapsulated *E. durans* IW3 was released from the prepared beads of ALG after 90 min, whereas both probiotics encapsulated in ALG‐GA and ALG‐PSY were released after 60 min. *Enterococcus durans* IW3 was successfully encapsulated in ALG, ALG‐GA, and ALG‐PSY beads prepared by extrusion method. ALG‐GA and ALG‐PSY beads are suitable delivery carriers for the oral administration of bioactive compounds like probiotics. The GA and PSY gels exhibited better potential for encapsulation of probiotic bacteria cells because of the amendment of ALG difficulties and utilization of therapeutic and prebiotic potentials of these herbal biopolymers.

## 1 Introduction

Encapsulation is the process of entrapping bioactive molecules (e.g., vitamins, minerals, antioxidants, fatty acid, lutein, and lycopene) and living cells (e.g., probiotics) within carrier materials (Nedovic, Kalusevic, Manojlovic, Levic, & Bugarski, 2011). Encapsulation is the most effective technique to protect probiotic bacteria during processing and storage (Kanmani et al., 2011). Improving the delivery of these active agents into food and medicine is important, and many substances can be used for encapsulation. Encapsulation materials are selected based on the following criteria: functionality, stability, type of release, encapsulates concentration, and cost. In addition, carrier materials must be biodegradable, biocompatible, food grade, and capable of barrier formation (Nedovic et al., 2011).

Encapsulation of probiotics causes the production of a physical barrier between the internal phase and its surrounding to protect them against pH alterations, moisture variations, and oxidation; thus, this process controls the release of active molecules and increases their bioavailability (Dubey, Shami, & Bhasker, 2009). The most significant aim for the encapsulation of active agents is to improve the stability of the final product. For instance, encapsulation can increase the bioavailability and functionality of probiotics (Milanovic et al., 2010; Shi et al., 2013), which are highly sensitive to the transport conditions, digestive enzymes, pH, and mechanical stress in the stomach. Encapsulation can cover the bitter taste of some food products by inhibiting reactions with other components, such as water and oxygen (Nedovic et al., 2011).

Recent research on biocompatible and biodegradable polymers has attracted considerable attention because of environmental concerns. The most extensively used materials for various encapsulations are polysaccharides, followed by proteins and lipids (Nesterenko, Alric, Violleau, Silvestre, & Durrieu, 2013). Examples of these polysaccharides are starch and its derivatives (e.g., amylose, dextrin, amylopectin, polydextrose, maltodextrins, and syrups), cellulose and its derivatives, plant exudates and extracts (e.g., gum Arabic [GA], mesquite gum, gum karaya, gum tragacanth, pectins, galactomannans, and soluble soybean polysaccharides), and marine extracts (e.g., carrageenan and alginate [ALG]) (Nedovic et al., 2011).

The most common encapsulation material is sodium ALG because of its simplicity, biocompatibility, non‐toxicity, and cost‐efficiency (Krasaekoopt et al. 2003). ALG, a polysaccharide extracted from algae, consists of β‐d‐mannuronic and α‐l‐guluronic acids. The various amounts and sequential distribution of β‐d‐mannuronic and α‐l‐guluronic acids in chain can affect the ALG functional properties as a supporting material (Burgain et al. 2011). However, ALG can provide limited protection to probiotics because of its notable properties. For example, ALG beads are not stable in acidic environment (Mortazavian et al. 2008). In addition, ALG microspheres with porous structure allow easy diffusion of acid in and out of microspheres. These disadvantages can be effectively overcome by blending ALG with other polymers or coating one polymer layer on ALG microspheres (Burgain et al. 2011).

New biopolymers for encapsulation purposes have been recently reported (Mahfoudhi et al., 2014). Gum exudates are predominantly composed of polysaccharides that function as stabilizing and emulsifying agents. GA has the highest commercial value among the gum exudates because of its extensive application in the pharmaceutical, food, and cosmetic industries (Mahfoudhi et al., 2014). GA, a natural polymer harvested from the branches and stems of *Acacia Senegal* trees, is highly used as exudates of water‐soluble gum. GA functions as a thickening agent, stabilizer, and hydrocolloid emulsifier without causing adverse effects because GA is a high‐molecular‐weight polysaccharide (Ali, Ziada, & Blunden, 2009; Johnson, 2005). GA is used as a carrier in the encapsulation of oils and other bioactive molecules (Karaiskou, Blekas, & Paraskevopoulou, 2008; Lambert, Weinbreck, & Kleerebezem, 2008) because of its biocompatibility for *in vivo* applications (Almuslet, Hassan, Al‐Sherbini, & Muhgoub, 2012). GA has the added benefits of providing the health profits associated with dietary fiber (Bliss et al. 2001), showing antibacterial activity against periodontal pathogens (Clark et al. 1994) and causing a rapid change in fecal flora when provided as part of the human diet. Desmond (2002) used spray‐dried powders containing GA to improve the survival of *Lactobacillus paracasei* NFBC 338. They showed that GA can protect probiotic bacteria during drying, storage, and gastric transit. This finding demonstrated that GA treatment of the probiotic‐containing powder results in efficient probiotic delivery to the gastrointestinal tract (GIT).

Psyllium (PSY), an arabinoxylan herbal‐based biopolymer, is extracted from *Plantago* species. PSY can stimulate the growth of probiotic bacteria in the GIT and treat several gut disorders, including ulcerative colitis, chronic kidney, constipation, and diarrhea (Guo, Cui, Wang, & Christopher Young, 2008; Rishniw & Wynn, 2011).

Enterococci are non‐spore‐forming, cocci‐shaped, gram‐positive, and catalase‐negative bacteria. These facultative anaerobic organisms may appear singly, in pairs, or in short chains (Nami, Haghshenas, Haghshenas, & Yari Khosroushahi, 2015). Enterococci thrive in the female genitourinary tract, particularly in the vagina, and the GIT (gut or bowel) without causing any infection (Nami et al., 2014). In this study, the probiotic strain *Enterococcus durans* IW3, isolated and identified from the Iranian traditional yogurt ecosystem, was selected for encapsulation because of its low cell viability at harsh acidic/bile conditions. This study has been conducted to evaluate the suitability of ALG, GA, and PSY to increase the viability of *E. durans* IW3 under industrial yogurt production conditions. The encapsulation efficiency of these biopolymers was also determined.

## 2 Materials and Methods

### 2.1 Bacterial strains and culture conditions

Probiotic strain *E. durans* IW3 isolated and identified from the Iranian traditional yogurt ecosystem was selected for encapsulation using natural‐based gels because of its low cell viability at low pH and under high bile salt condition. The isolated strain was grown on MRS medium (Merck, Germany) under anaerobic conditions at 37°C for 18–24 hr. Cells in the late‐log phase (2 × 10^9^ CFU/g) were harvested by centrifugation at 700 *g* for 10 min at 4°C. The cells were washed and resuspended in phosphate buffer saline (PBS; pH 7.4, 10 mmol/L $PO_{4}^{-3}$, 137 mmol/L NaCl, and 2.7 mmol/L KCl) under the same centrifugation conditions and then counted thrice on MRS agar using the pour plate technique. An equal volume with the same viable cell population was divided for use in encapsulation by different herbal‐based gels.

### 2.2 Isolation, molecular identification, and characterization of *E. durans* IW3

*Enterococcus durans* IW3 was isolated from 60 samples of traditional yogurt that were randomly collected from the retailers in different parts of Kermanshah province in Iran. This probiotic species was isolated and amplified through anaerobical growth of MRS broth medium for 24 hr at 37°C and spread on MRS agar media similar to mentioned condition (Mirzaei & Barzgari, 2012). The total genomic DNA was extracted by the method described by Leenhouts, Kok, and Venema (1990) with some modifications. The 16S‐rDNA gene amplification was carried using the universal bacterial primer pairs namely, F: 5′‐AGAGTTTGATCMTGGCTCAG‐3′ and R: 5′‐TACCTTGTTAGGACTTCACC‐3′. The PCR program cycles were as follows: denaturation at 95°C for 4 min, 32 cycle of: 94°C, 1 min, 58°C, 1 min, 72°C for 95 s, and the final extension was performed for 5 min in 72°C. The PCR‐amplified 1,500 bp fragment of 16S‐rDNA gene of this isolate was isolated from traditional yogurt and was sequenced and blasted with the deposited sequences in GenBank site (http://blast.ncbi.nlm.nih.gov/Blast.cgi). Isolate with 99–100% homology was identified as *E. durans* IW3 by considering the threshold values of taxonomical studies (97%) (Deng, Xi, Mao, & Wanapat, 2008).

### 2.3 GA aqueous solution preparation

The pharmaceutical grade of GA (Dingli Industry Garden, Tai'an, Shandong, China) was purchased from a local shop in Tabriz, Iran and was used without further purification. A 10% aqueous solution of GA was prepared by completely dissolving 10 g of gum dried powder in 100 ml distilled water by rapid mechanical stirring. The solution was stored at room temperature for 3–5 hr and then diluted to different concentrations.

### 2.4 PSY solution preparation

The pharmaceutical grade of PSY husk (Altrafine Gums, Vatva, Ahmedabad, India) was used to prepare the PSY aqueous solution based from previously described method by Guo et al. (2008) with slight modifications. Afterward, 10 g of PSY husk was added to 200 ml of hot water (80°C). A homogenous gel solution was produced after 18 hr of gentle mixing. The solution was centrifuged at 14,000 *g* for 30 min to separate the gel from the aqueous phase. The separated gel was dissolved in NaOH solution (2 mol/L) by 2 hr of incubation at 37°C. An alkaline gel solution was produced after 30 min of centrifugation at 14,000 *g* and neutralized by adding HCl solution (2 mol/L). Finally, a yellow gel was separated from the solution after 30 min of centrifugation at 14,000 *g* and washed twice with distilled water.

### 2.5 ALG‐based bead preparation by extrusion

*Enterococcus durans* IW3 capsules with pharmaceutical grade ALG (sodium salt; Sigma‐Aldrich, Taufkirchen, Germany), ALG‐GA, and ALG‐PSY were prepared through extrusion. Na‐ALG at the concentration range of 0.75–2% (w/v) is the most prevalent biopolymer in probiotic encapsulation (Chandramouli, Kailasapathy, Peiris, & Jones, 2004). The use of high‐concentration [>2% (w/v)] biopolymer matrices as carriers is difficult because their high viscosity complicates their extrusion through a syringe or nozzle. Moreover, the use of low‐concentration biopolymer matrices [<1% (w/v)] is difficult because their low viscosity and cross‐linking site formation prevent them from creating uniform encapsulated beads (Lotfipour, Mirzaeei, & Maghsoodi, 2012).

Na‐ALG, GA, and PSY solutions were prepared in one batch with different concentrations (Table 1) by dissolving in distilled water (Lotfipour et al., 2012). The solutions were filtered through a 0.2 μm membrane filter (GH Polypro; PALL Gelman Laboratory; Lund, Sweden) to sterilize them. To prepare a homogenous solution, 10% (w/v) of the probiotics was added to each solution of the polymers (ALG, ALG‐GA, and ALG‐PSY) and then stirred for 30 min. The solutions were extruded into 0.5 mol/L CaCl_2_ sterile solution (100 ml) by using 21‐gage nozzles, gently homogenized at 250 rpm, and then filtered (Whatman No. 1) to form beads that contained *E. durans*. The beads were washed thrice with distilled water and then stored in 0.1% (w/v) peptone solution (Sigma–Aldrich) at 4°C (Albertini et al., 2010).

**Table 1:** Compositions and the size of prepared beads in each formulation

Formulation	ALG (% w/v)	PSY/GA (% w/v)	ALG‐GA diameter (mm) (*n* = 50)^a^	ALG‐PSY diameter (mm) (*n* = 50)
F1	2	–	0.98 ± 0.05	0.98 ± 0.03
F2	2	0.1	1.28 ± 0.08	1.15 ± 0.09
F3	2	0.3	1.50 ± 0.03	1.23 ± 0.05
F4	1.5	–	0.76 ± 0.05	0.76 ± 0.07
F5	1.5	0.3	0.84 ± 0.06	0.79 ± 0.04
F6	1.5	0.5	0.97 ± 0.09	0.94 ± 0.08
F7	1	–	0.33 ± 0.07	0.33 ± 0.04
F8	1	0.4	0.51 ± 0.04	0.46 ± 0.04
F9	1	0.6	0.62 ± 0.08	0.59 ± 0.06

a Each diameter is the average size of 50 beads.

### 2.6 Free and encapsulated bacterial enumeration

*Enterococcus durans* IW3 was enumerated on MRS agar by using the pour plate method. To count the number of viable cells of free probiotic strains, 1 g of pellet of *E. durans* IW3 was added to 100 ml of PBS (pH 7.4) to be homogenized, and then they were incubated at 37°C for 30 min. The probiotic sample was serially diluted and pour‐plated on MRS agar and then anaerobically incubated at 37°C for 18–36 hr. Counts were expressed as number of CFU per gram of product. The data were expressed as the mean of the three counts ± standard error.

Encapsulated *E. durans* IW3 was retained in industrial yogurt condition by gentle shaking at room temperature until completely released. The released *E. durans* IW3 solutions were serially diluted 10 times in PBS solution. The survival rates of *E. durans* IW3 released from the beads were immediately evaluated by plating on MRS agar.

### 2.7 Encapsulation efficiency

Encapsulation efficiency (EE) was determined by disintegrating the encapsulated bacteria particles in phosphate buffer (pH 7.4, 10 mmol/L $PO_{4}^{-3}$, 137 mmol/L NaCl, and 2.7 mmol/L KCl). In brief, 50 mg of each encapsulated beads was disintegrated in 10 ml phosphate buffer (pH 7.4) at 37°C for 30 min. Subsequently, the entrapped viable bacteria were counted by the pour plate technique in MRS agar. In pour plate method, the probiotic samples were serially diluted and pour‐plated on MRS agar and then anaerobically incubated at 37°C for 24 hr. Counts were expressed as number of CFU per gram of product. EE was calculated using the following formula (Lotfipour et al., 2012):

$$\text{EE}=({\text{log}}_{10}N/{\text{log}}_{10}N_{0})\times 100$$

where *N* is the number viable bacteria (CFU) entrapped by biopolymers, and *N*_0_ is the number of free viable bacteria before encapsulation.

### 2.8 Morphological analysis

The size and topographical properties of the bacterial beads were determined through Olympus BX61 optical microscopy (Olympus, Tokyo, Japan). The mean size of 50 beads for each gel formulation was investigated.

### 2.9 Moisture content and water activity of microspheres

The moisture content of powdered microspheres was evaluated according to a modified method by Eratte et al. (2015). Samples were dried in an oven at 105°C for 24 hr. The water activity of microspheres was determined using a water activity meter (Aqualab CX2, Decagon Devices, Washington) at maintained temperature (24 ± 0.5°C).

### 2.10 Viability of encapsulated and free bacteria at low pH and high bile salt concentration

Approximately 100 mg of the beads was mixed by gentle agitation and then incubated in 20 ml of a low pH of PBS solution (pH 2.0) (Haghshenas et al., 2015; Picot & Lacroix, 2004) for 2 hr at 37°C to assess the survival rate of the encapsulated and free probiotic bacteria. Phosphate buffer (pH 7.4) was used to disintegrate the treated beads. The CFU of the bacterial cells was counted on MRS agar using the pour plate technique.

The viability of the encapsulated *E. durans* IW3 at high bile salt solution was evaluated according to the method described by Ma et al. (2008). The encapsulated probiotic products were transferred into 1 g/100 ml of bile salt solution (0.5% w/v oxgall; Merck, Germany) and then incubated at 37°C with gentle shaking at pH 7.4 for 2 hr. The number of viable cells was counted following the procedure described in the section of “Free and encapsulated bacterial enumeration.” Finally, the survival rate (%) of the bacteria was measured as follows:

Survival rate(%)=(log CFU/g capsules after treatment/log CFU/g capsules before treatment)×100.

### 2.11 Storage stability of encapsulated *E. durans* IW3 in yogurt

The storage stability was carried out according to a modified technique described by Shi et al. (2013). The stability of non‐encapsulated and encapsulated bacteria was assessed during 1‐month storage in yogurt at 4°C. The viability of cells in seven different storage times (0, 5, 10, 15, 20, 25, and 30 days) was determined. During storage time, 0.5 g encapsulated cells at the room temperature by gentle shaking (100 rpm) was dissolved in 5 ml sodium citrate solution (50 mmol/L) with pH 7.5. The released and non‐encapsulated probiotic cells were serially diluted 10 times using saline solution, and then, 50 μl of aliquots was placed on the MRS agar for 24 hr anaerobic growth (37°C). The viable (%) rates of probiotic cells were calculated by using the pour plate technique in MRS agar. Meanwhile, acidity of yogurt containing free and encapsulated probiotic cells was measured during storage time.

### 2.12 Release study of encapsulated *E. durans* IW3

Release profile of encapsulated *E. durans* IW3 in different biopolymer beads in yogurt was studied. Different yogurts possessing 10% biopolymer beads containing *E. durans* IW3 (500 mg) were added to tubes containing prewarmed simulated intestine fluid, SIF, (pH 6.8, 50 mmol/L KH_2_PO_4_; Ricca Chemical Company, TX) and incubated at 37°C with gentle shaking at 100*g*. The bacterial beads were transferred into SIF and then incubated at 37°C with gentle shaking at 100 ×g. At predetermined intervals, the picked‐up samples were filtered through 5 μm Acrodisc^®^ syringe filters (Gelman, Pall Corporation, Ann Arbor, MI) to separate the released bacteria from the beads (mm size in diameter). Afterward, 100 μl aliquots was taken out and immediately assayed for the amounts of released *E. durans* IW3. Finally, the viable counts of bacteria were determined by plating on MRS agar plates at 37°C for 48 hr (Haghshenas et al., 2015). The percentage of bacteria released from beads at predetermined intervals was calculated based on the equation below:

Percentage of release(%)=(CFU of released bacteria/total CFU of bacteria loaded in beads)×100.

### 2.13 Statistical analyses

Statistical differences between the experiments were determined by ANOVA with a confidence interval of 95%. Significant differences among treatment means were tested by the Duncan multiple comparisons test using SPSS 19.0 at *p* ≤ .05. Each experiment was repeated in triplicates (*n* = 3).

## 3 Results

### 3.1 Morphology, size, and EE of the produced beads

Based on our findings, the mean diameters of 50 ALG, ALG‐GA, and ALG‐PSY spheres prepared using 21‐gage needle were 690 μm (range: 330–980 μm), 953 μm (range: 510 μm to 1.5 mm), and 860 μm (range: 460 μm to 1.23 mm), respectively. The mean diameters of the encapsulated particles with GA and PSY were significantly higher than those without GA and PSY (*p* ≤ .05).

In this research, bead sizes increased when the total biopolymer concentration increased. For instance, the sizes of ALG‐GA and ALG‐PSY beads increased from 0.51 ± 0.04 mm to 1.50 ± 0.03 mm and 0.46 ± 0.04 mm to 1.23 ± 0.05 mm, respectively (Table 1). The bead sizes increased when the ALG concentration exceeded from 0.33 ± 0.07 mm to 0.98 ± 0.05 mm.

Table 2 presents the EE for *E. durans* IW3 by using different biopolymer matrices ranging from 98.6% to 99.78%. No significant differences (*p* ≤ .05) existed between all these gel formulations.

**Table 2:** Mean count (log CFU/g) before and after encapsulation and encapsulation efficiency (%) of *Enterococcus durans* IW3 with different prepared biopolymeric matrices

Encapsulation formulation	Mean count before encapsulation (log CFU/g)	Mean count after encapsulation (log CFU/g)	Encapsulation efficiency (%)
*E. durans* IW3 + F1	9.15 ± 0.07	9.03 ± 0.07	98.69^a^
*E. durans* IW3 + F2, PSY	9.29 ± 0.01	9.16 ± 0.06	98.60^a^
*E. durans* IW3 + F2, GA	9.30 ± 0.04	9.18 ± 0.03	98.72^a^
*E. durans* IW3 + F3, PSY	9.25 ± 0.06	9.16 ± 0.05	99.03^a^
*E. durans* IW3 + F3, GA	9.27 ± 0.04	9.19 ± 0.07	99.14^a^
*E. durans* IW3 + F4	9.22 ± 0.02	9.11 ± 0.04	98.81^a^
*E. durans* IW3 + F5, PSY	9.68 ± 0.02	9.58 ± 0.01	98.97^a^
*E. durans* IW3 + F5, GA	9.58 ± 0.03	9.51 ± 0.09	99.27^a^
*E. durans* IW3 + F6, PSY	9.49 ± 0.07	9.41 ± 0.03	99.16^a^
*E. durans* IW3 + F6, GA	9.42 ± 0.02	9.39 ± 0.06	99.68^a^
*E. durans* IW3 + F7	9.30 ± 0.05	9.24 ± 0.08	99.35^a^
*E. durans* IW3 + F8, PSY	9.27 ± 0.05	9.24 ± 0.02	99.67^a^
*E. durans* IW3 + F8, GA	9.34 ± 0.09	9.32 ± 0.05	99.78^a^
*E. durans* IW3 + F9, PSY	9.50 ± 0.06	9.46 ± 0.07	99.58^a^
*E. durans* IW3 + F9, GA	9.20 ± 0.08	9.18 ± 0.02	99.78^a^

a Means are not significantly different (*p < *.05).

### 3.2 Moisture content and water activity of microspheres

The physicochemical characteristics (moisture content and water activity) of microspheres prepared by various formulations are discussed in this section. The moisture content of all prepared formulations was lower than 3.23% (w/w). There were no significant differences in moisture content of these nine gel formulations and control (ALG). The water activity values of alginate‐Gum Arabic blends (F2, F3, F4, and F5) were significantly (*p* ≤ .05) lower than other formulations. On the other hand, control (ALG) showed the highest water activity value (*p* ≤ .05) than other blends (Table 3).

**Table 3:** Formulations, compositions, moisture content (%), and water activity of encapsulated *E. durans* IW3 with various prebiotic concentrations. Alginate‐encapsulated cells (2% (w/v)) were used as control. F1–F9: various gel formulations. Values shown are means ± standard deviations (*n* = 3)

Formulations	Prebiotics	Con (%)	Moisture content (%)	Water activity
F1 (ALG)	–	–	3.23 ± 0.3^a^	0.46 ± 0.02^a^
F2	Psyllium	0.5	3.03 ± 0.2^a^	0.18 ± 0.02^a^
F3	Psyllium	1.0	3.11 ± 0.4^a^	0.17 ± 0.03^a^
F4	Psyllium	1.5	3.19 ± 0.2^a^	0.15 ± 0.04^a^
F5	Psyllium	2.0	3.14 ± 0.4^a^	0.16 ± 0.03^a^
F6	Gum Arabic	0.5	3.02 ± 0.3^a^	0.30 ± 0.04^a^
F7	Gum Arabic	1.0	2.99 ± 0.2^a^	0.29 ± 0.03^a^
F8	Gum Arabic	1.5	3.05 ± 0.4^a^	0.29 ± 0.02^a^
F9	Gum Arabic	2.0	3.03 ± 0.3^a^	0.27 ± 0.03^a^

a Values followed by the same letters are not significantly different (*p* > .05). Statistical analysis of each formulation was performed separately. ALG: alginate‐encapsulated cells, Con: concentration.

### 3.3 Viability of encapsulated and free bacteria at low pH

This probiotic strain was highly sensitive to low pH. The viability of all free *E. durans* IW3 significantly decreased after exposure to acidic conditions. As shown in Table 4, the cell counts of the untreated *E. durans* IW3 decreased from 9.38 ± 0.03 log CFU/g to 4.05 ± 0.01 log CFU/g after 2 hr exposure to acidic conditions. These results indicate 43% survival rate of *E. durans* IW3.

**Table 4:** Mean count of *Enterococcus durans* IW3 before and after low pH treatment (pH 2.0) and their survival rates in different prepared formulations

Encapsulation formula	Mean count before low pH treatment (log CFU/g)	Mean count after low pH treatment (log CFU/g)	Survival rate (%)		
*E. durans* IW3	9.38 ± 0.03	4.05 ± 0.01	43^a^		
IW3 + F1	9.28 ± 0.07	7.81 ± 0.04	84^a^	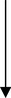	
IW3 + F2, PSY	9.34 ± 0.02	8.26 ± 0.07	88^a^		
IW3 + F2, GA	9.29 ± 0.08	8.30 ± 0.04	89^a^		
IW3 + F3, PSY	9.45 ± 0.03	8.63 ± 0.08	91^a^		
IW3 + F3, GA	9.32 ± 0.02	8.70 ± 0.06	93^a^		
IW3 + F4	9.27 ± 0.07	7.18 ± 0.01	78^a^		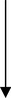
IW3 + F5, PSY	9.40 ± 0.04	7.67 ± 0.07	81^a^		
IW3 + F5, GA	9.43 ± 0.08	7.75 ± 0.05	82^a^		
IW3 + F6, PSY	9.38 ± 0.03	7.93 ± 0.03	84^a^		
IW3 + F6, GA	9.36 ± 0.01	7.97 ± 0.06	85^a^		
IW3 + F7	9.39 ± 0.05	7.17 ± 0.01	76^a^	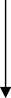	
IW3 + F8, PSY	9.25 ± 0.03	7.40 ± 0.03	80^a^		
IW3 + F8, GA	9.41 ± 0.08	7.62 ± 0.07	81^a^		
IW3 + F9, PSY	9.31 ± 0.02	7.61 ± 0.02	82^a^		
IW3 + F9, GA	9.35 ± 0.07	7.71 ± 0.08	82^a^		

a Means with the same letter are not significantly different (*p *< .05).

Note. The arrow shows that letters a, b, and c show the significant means.

The cell counts of *E. durans* IW3 encapsulated in 2% ALG decreased from 9.28 ± 0.07 log CFU/g to 7.81 ± 0.04 log CFU/g after 2 hr exposure to acidic conditions. These results indicate approximately 84% survival rate of *E. durans* IW3. In addition, the cell counts of *E. durans* IW3 encapsulated in 1% ALG decreased from 9.39 ± 0.05 log CFU/g to 7.17 ± 0.01 log CFU/g after 2 hr exposure to acidic conditions. These results indicate approximately 76% survival rate of *E. durans* IW3. The survival rates of *E. durans* IW3 in 1%, 1.5%, and 2% ALG were 76%, 78%, and 84%, respectively. These results indicate that the high percent of ALG (2%) is more capable to retain the survivability of probiotics than the low percent (1%).

All prepared beads significantly increased the survival rates of the probiotic bacteria after acid exposure (*p* ≤ .05). The cell counts of the encapsulated *E. durans* IW3 decreased to 2.22 log CFU/g, whereas those of the free bacteria decreased to 5.33 log CFU/g after 2 hr exposure to acidic conditions. These results confirmed that the biopolymer matrices, ALG, ALG‐GA, and ALG‐PSY, enhanced the viability of *E. durans* IW3 under low pH conditions. Encapsulation with all biopolymer matrices increased the survival rates of *E. durans* IW3 by 33–50%. These results show that all biopolymers showed good protective effects on *E. durans*.

The survival rate of *E. durans* IW3 under low pH conditions was increased by incorporating GA and PSY into ALG. As shown in Table 4, the survival rates of *E. durans* IW3 encapsulated in ALG‐PSY and ALG‐GA slightly increased by increasing the percent of integrated PSY and GA. By contrast, integrating GA to ALG showed a better result than integrating the same amount of PSY. These results demonstrated that the survival rate of probiotics under low pH conditions depended on the formulation of the carrier and on the incorporation of herbal‐based biopolymers, such as GA and PSY, into ALG. Formulation 3 showed the best results, whereas formulation 7 showed the worst results.

### 3.4 Viability of encapsulated and free probiotics at high bile salt concentration

Table 5 presents the survival rates of the free and encapsulated *E. durans* IW3 after 2 hr exposure to 0.5% (w/v) oxgall. In this study, the viability rate of the free *E. durans* IW3 significantly decreased after 2 hr exposure to 0.5% (w/v) oxgall. This result can be attributed to the damaging effects of oxgall on cell wall integrity. Many studies revealed that probiotics are sensitive to bile salt solution. For instance, approximately 2 and 5 log CFU/ml reductions were observed in the survival rate of *Bifidobacterium adolescentis* after 2 hr exposure to 0.5% (Truelstup‐Hansen, Allan‐Wojtas, Jin, & Paulson, 2002) and 2% (w/v) bile salt solutions (Clark & Martin, 1994) at 37°C, respectively.

**Table 5:** Mean count (log CFU/g) of *Enterococcus durans* IW3 before and after bile salt treatment (0.5% oxgall) and their survival rates in prepared formulations

Encapsulation formula	Mean count before bile salt treatment (log CFU/g)	Mean count after bile salt treatment (log CFU/g)	Survival rate (%)		
*E. durans IW3*	9.39 ± 0.04	5.51 ± 0.08	59^a^		
IW3 + F1	9.32 ± 0.07	8.06 ± 0.03	86^a^	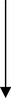	
IW3 + F2, PSY	9.41 ± 0.02	8.69 ± 0.04	92^a^		
IW3 + F2, GA	9.37 ± 0.06	8.70 ± 0.06	93^a^		
IW3 + F3, PSY	9.29 ± 0.03	8.81 ± 0.05	95^a^		
IW3 + F3, GA	9.26 ± 0.04	8.81 ± 0.02	95^a^		
IW3 + F4	9.42 ± 0.02	7.85 ± 0.07	83^a^		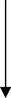
IW3 + F5, PSY	9.38 ± 0.08	8.38 ± 0.06	89^a^		
IW3 + F5, GA	9.33 ± 0.03	8.37 ± 0.04	90^a^		
IW3 + F6, PSY	9.45 ± 0.08	8.59 ± 0.03	91^a^		
IW3 + F6, GA	9.31 ± 0.07	8.51 ± 0.08	91^a^		
IW3 + F7	9.38 ± 0.01	7.58 ± 0.07	81^a^	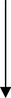	
IW3 + F8, PSY	9.34 ± 0.06	8.06 ± 0.04	86^a^		
IW3 + F8, GA	9.30 ± 0.05	8.09 ± 0.02	87^a^		
IW3 + F9, PSY	9.42 ± 0.01	8.35 ± 0.08	88^a^		
IW3 + F9, GA	9.40 ± 0.09	8.36 ± 0.05	89^a^		

a Means with the same letter are not significantly different (*p *< .05).

Note. The arrow shows that letters a, b, and c show the significant means.

However, Table 5 evidently shows that the ALG, ALG‐GA, and ALG‐PSY beads can provide significant protection against bile salt (*p* ≤ .05). The viable counts of *E. durans* IW3 encapsulated in 2%, 1.5%, and 1% ALG decreased from 9.38 ± 0.01 log CFU/g to 7.58 ± 0.07 log CFU/g, 9.42 ± 0.02 log CFU/g to 7.58 ± 0.07 log CFU/g, and 9.32 ± 0.07 log CFU/g to 8.06 ± 0.03 log CFU/g beads after 2 hr exposure to 0.5% bile salt solution, respectively. *Enterococcus durans* IW3 encapsulated in ALG beads demonstrated better survivability (less than 1.8 log reduction) after 2 hr bile incubation compared with free *E. durans* IW3 (*p* ≤ .05), which showed approximately 3.88 log CFU/g reduction, after 2 hr exposure to 0.5% bile salt solution.

### 3.5 Release of encapsulated beads in SIF

**Figure 1:**
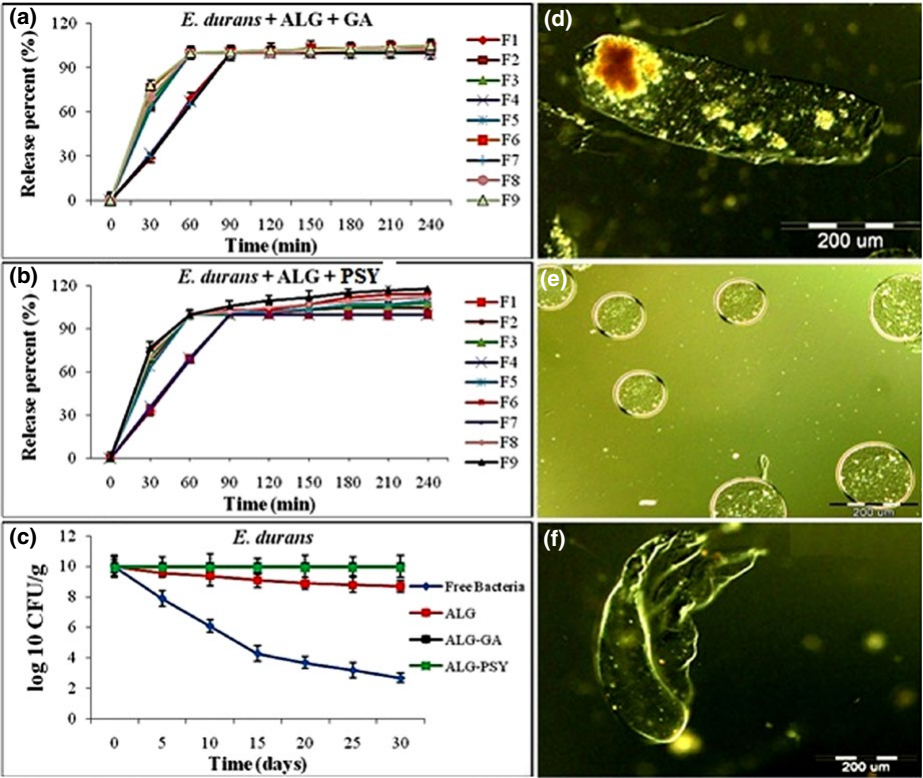
(a and b) Release profiles of encapsulated *Enterococcus durans* IW3 in SIF pH 6.8. Values presented are means ± standard deviations (*n* = 3). (c) Storage stability of free and encapsulated *E. durans* IW3 in yogurt at 4°C during 1 month. Conditions: ALG concentration 2%, GA and PSY concentration 0.6%. Values shown are means ± standard deviations (*n* = 3). (e and f) represents the optical images of encapsulated beads by the optical microscopy (Olympus BX61): (d) alginate‐gum Arabic blend, (e) alginate‐psyllium blend, and (f) alginate

Figure 1 shows the release characteristics of encapsulated probiotics in SIF. The ALG‐GA and ALG‐PSY beads had faster released rate than the ALG beads in SIF. All encapsulated *E. durans* IW3 was released from the prepared beads of ALG after 90 min, whereas both probiotics encapsulated in ALG‐GA and ALG‐PSY were released after 60 min. These results indicated that the encapsulation with ALG‐GA and ALG‐PSY peaked after 60 min, yet the encapsulation with ALG peaked after 90 min.

### 3.6 Storage stability of free and encapsulated bacteria in yogurt

Microencapsulated beads (10%) were added into yogurt on the day of their preparation. The probiotics in yogurt were enumerated periodically after 1 day for 1 day intervals in the cold room until one month. Storage stability test was performed at 4°C to investigate the efficiency of encapsulation to reduce the loss of probiotics viability. Figure 1 illustrates the storage stability of free and encapsulated *E. durans* IW3 in ALG (2% w/v), ALG‐GA (2% + 0.6%), and ALG‐PSY (2% + 0.6%) in yogurt at 4°C for 30 days. Viability of encapsulated *E. durans* IW3 in ALG‐GA‐ and ALG‐PSY‐based beads could be completely preserved. However, the survival counts of encapsulated *E. durans* IW3 in ALG and free *E. durans* IW3 reduced from 10 log CFU/g to 8.7 log CFU/g and 2.7 log CFU/g after 30 days of storage, respectively. The highest rates of decrease were found in the first 15‐days while in the second 15‐days, a decrease with the low slope was observed. This probably was due to temperature shock in the first 15‐days and the subsequent adaptation process (Figure 1c).

## 4 Discussion

According to our results and FAO/WHO guidelines, identification of Enterococcus strains by sequencing of 16S‐rDNA can be considered as an accessible and suitable technique. The threshold value for taxonomical studies is around 97%, hence, 16S‐rDNA sequencing with 99–100% homology was performed for phylogenetic clustering as a valid and accurate technique (Deng et al., 2008).

Extrusion technique was selected to encapsulate *E. durans* IW3 in an aqueous solution because of its mildness, low cost, high viability rates, and easy performance (Ma et al. 2012; Sohail, Turner, Coombes, Bostrom, & Bhandari, 2011). Extrusion is the most common encapsulation technique for probiotic bacteria. The size and EE of beads produced by extrusion are affected by many factors, including biopolymer concentration and composition, nozzle size, and distance between nozzle and setting bath. This size can vary between 200 μm and 5 mm (Voo, Ravindra, Tey, & Chan, 2011).

The combination of PSY and GA into ALG gel increased the viscosity and adherence of the resultant gel. PSY gel added in the blend may affect the viscosity and exceeded the ability of extrusion method for spherical bead formation. These results are consistent with other research in extrusion method; the decrease in the viscosity of supporting gels leads to the preparation of smaller beads (Kailasapathy, 2002; Lotfipour et al., 2012).

Biopolymer concentration can cause a large variation in sphere size. In this research, bead sizes increased when the total biopolymer concentration increased. ALG concentrations in beads affected not only bead sizes but also the sphericity and flexibility of beads. The bead sizes significantly increased (*p* ≤ .05) when the ALG concentration increased in the beads (Table 1). Beads produced by higher percent of biopolymers had larger diameters compared with those prepared using lower percent of biopolymers. ALG encapsulation can be affected by a range of factors such as probiotic cell load, ALG concentration, capsule size, and hardening time in calcium chloride.

Therefore, the encapsulation efficiency was formulation‐independent. However, some references reported that the polymer composition and concentration can affect the encapsulation efficiency. EE, an important parameter of encapsulation, is influenced by the encapsulation procedure and the chemical nature of both encapsulating biopolymers and content (Cheng, Liu, & He, 2010). Previous studies reported that biopolymer concentration can affect the EE. However, different biopolymer concentrations did not show significant differences in EE in this study. Shi et al. (2013) reported that the increase of milk in ALG beads can improve the EE of *Lactobacillus bulgaricus*. Beads with large size provide more protection than those with small size. In this study, beads with large size (formulation 3) provided more protection than those with small size (formulation 7).

The physicochemical characteristics (moisture content and water activity) of microspheres prepared by various formulations are discussed in this section. Eratte et al. (2015) and Gardiner et al. (2000) observed the low content of moisture and water activity was same as our results during microencapsulation of probiotics. It was reported that low residual water contents and water activity can improve the storability and stability of powdered‐beads containing probiotic bacteria.

Probiotics should resist the stressful conditions of the GIT to exert their beneficial health effects. Encapsulation is performed to improve the low pH tolerance of probiotics. The pH range of gastric juices is approximately 1.5–3.0 (Kos, Suskovic, Goreta, & Matosic, 2000). In this paper, 2 hr of incubation with a pH 2.0 solution was performed. Table 4 shows the pH stabilities and survival rates of the free and encapsulated *E. durans* IW3 with different biopolymer matrices. Our findings are similar to studies that evaluated the capability of ALG coating to protect probiotic bacteria under acidic conditions (Kim et al., 2008; Shi et al., 2013). For instance, Mokarram et al. (2009) demonstrated the efficacy of multistage ALG coating on increasing the survival rates of probiotics in simulated gastrointestinal juices. Furthermore, Sohail et al. (2011) reported that the encapsulation in cross‐linked ALG microspheres can increase the survival rates of probiotics under harsh gastrointestinal conditions. Conversely, Sultana et al. (2000) revealed that the encapsulation in ALG beads cannot efficiently protect bacteria from high acidity.

The integration of PSY and GA into ALG beads slightly increased the viability rate of *E. durans* IW3 under acidic conditions. PSY and ALG combination increases the survival rate of *Lactobacillus acidophilus* in a PSY dose‐dependent manner (Lotfipour et al., 2012). This type of compositions is extensively used in the encapsulation of probiotic bacteria. Our results correspond well with the results of Lotfipour (2012). Albertini et al. (2010) reported that the combination of xanthan gum and ALG increases the survival rates of probiotics under acidic conditions. Furthermore, the incorporation of starch into ALG enhances the protection level of bacteria under acidic conditions (Muthukumarasamy, Allan‐Wojtas, & Holley, 2006).

According to results presented in Table 5, integration of PSY (0.3%) and GA (0.3%) into ALG (2%, F3) increased the survival rate of *E. durans* IW3 beads to 36.25% and 36. 57% compared with *E. durans* IW3 beads encapsulated in ALG (2%, F1) with increased survival rate of 27.94%. The result is possibly caused by the structured trapping ALG‐GA and ALG‐PSY matrices that are more resistant to the effects of bile salt solution. To compare our results to other research is difficult because researchers used different concentrations and sources of bile salts. In this study, the encapsulated cells showed higher tolerance to bile solution than the free cells. Encapsulated probiotic bacteria can survive better in 1–3% bile salt solution than free probiotic bacteria (Kailasapathy and Masondole 2005).

To elicit the beneficial effects of probiotics on the host, the bacteria must survive through the upper digestive tract to reach the large intestine where they are expected to proliferate and colonize (Shah, 2000). Encapsulated probiotics must be released in SIF before they can exert beneficial effects on the human body (Ma et al., 2008). Probiotics encapsulated in prepared beads can be released fast. Microbeads pass through the stomach to reach the intestine with high pH and release probiotics. Several studies reported that the probiotic bacteria encapsulated in ALG beads can be completely released and remained constant after 1 hr (Lotfipour et al., 2012; Mandal & Puniya, 2006).

In the case of beads prepared using ALG‐PSY, not only *E. durans* IW3 was completely released from the beads after 60 min but the diverse rates of bacterial growth were observed beyond the 60 min time. These results indicate the stimulating effect of PSY on the bacteria. Our results showed that higher concentrations of PSY produced greater stimulation effect on bacteria, as 0.6% w/v PSY in formulation 9 showed approximately 18% increase in *E. durans* IW3 count (see Figure 1). Similarly, the higher concentration of GA (0.6% w/v in F9) produced a little stimulation effect on bacteria (around 5% rise). By contrast, the lowest amounts of growth were observed in formulation 2 with minimum amount of GA and PSY (0.1% w/v). The stimulation effect of GA and PSY on *E. durans* IW3 may be attributed to its prebiotic properties and also its structures as soluble fibers. In some cases, PSY has been previously used as prebiotic (Damaskos & Kolios, 2008; Elli, Cattivelli, Soldi, Bonatti, & Morelli, 2008; Fujimori, Gudis, & Mitsui, 2009) and thus, supports the finding in our study.

The release mechanisms were possibly due to the swelling erosion of ALG‐GA and ALG‐PSY networks in SIF. Biopolymer composition and concentration not only influenced the protection of probiotic strains against acid and bile but also affected the release profile of encapsulated probiotics. The integration of GA and PSY into ALG caused the probiotics to be released faster from the beads containing GA and PSY. PSY, as a potential prebiotic, can improve the release and delivery of probiotic cells to the active sites and thus enhance the probiotic population in the colon. However, the release characteristic of encapsulated probiotics does not significantly change when the ALG concentration increases (Mandal & Puniya, 2006).

These results indicate that the encapsulation of *E. durans* IW3 in ALG, ALG‐GA, and ALG‐PSY can significantly improve the stability of *E. durans* IW3 in yogurt at 4°C. Incorporation of PSY and GA to ALG can improve ALG properties. This phenomenon is possibly caused by the prebiotic properties of PSY and GA and the formation of double layer structure in PSY‐based beads, which is shown in Figure 1c.

The probiotic carrier products such as yogurt usually are stored at the fridge temperature for one month; hence, the stability experiments were carried out in mentioned conditions (Brown et al., 2014; Casarotti et al., 2014). Free *E. durans* IW3 cells displayed a dramatic decrease in their cell viability during 1‐month storage at 4°C. The same result was found by Shi et al. (2013) where the cell viability of free *L. bulgaricus* after 1‐month storage dropped from 10 to 2.3 log CFU/g. Results indicated that microencapsulated cells in these gel formulations displayed significantly high viability at the storage time (*p* ≤ .05). The improvement of probiotic viability under refrigerated storage conditions was reported by many researchers. For instance, Shi et al. (2013) reported that carrageenan‐locust bean gum‐coated milk beads improve the stability of *L. bulgaricus* during the four weeks of storage. They also indicated that ALG‐milk sphere can improve the viability of *L. bulgaricus* during the 4‐week storage period. Several researchers reported that ALG‐chitosan and ALG‐human milk‐based beads can improve the storage stability of *Lactobacillus plantarum* and Bifidobacterium longum during the storage period (Chavarri et al., 2010; Cook, Tzortzis, Charalampopoulos, & Khutoryanskiy, 2011; Song, Yu, Liu, & Ma, 2014). Krasaekoopt (2003) reported that the encapsulation of probiotics with polymers, which blended with other polymers, can improve their storage stability.

Overall, *E. durans* IW3 was successfully encapsulated in ALG, ALG‐GA, and ALG‐PSY beads prepared by extrusion method. The viable cells of encapsulated *E. durans* IW3 in ALG‐GA and ALG‐PSY beads showed better survival ability than of those encapsulated in ALG and also free cells at low pH (pH 2.0), high bile salt concentration (0.5%), and longtime storage (30 days). Encapsulated *E. durans* IW3 in ALG‐GA and ALG‐PSY beads released in SIF had faster rate than those encapsulated in ALG beads. Encapsulation of *E. durans* IW3 using extrusion method has been proven as an appropriate method to protect from probiotics in food and gastrointestinal environments. ALG‐GA and ALG‐PSY beads are suitable delivery carriers for the oral administration of bioactive compounds like probiotics.

## Ethical Issues

No ethical issues were promulgated.

## Conflict of Interest

The authors declare no conflict of interests.
